# Association between body mass index (BMI) and vital capacity of college students of Zhuang nationality in China: a cross-section study

**DOI:** 10.18632/oncotarget.20758

**Published:** 2017-09-08

**Authors:** Peng Liu, Ziliang Ye, Haili Lu, Jingjing Lu, Liqian Huang, Jiangu Gong, Qiongying Deng, Lin Xu

**Affiliations:** ^1^ Department of Anatomy, Guangxi Medical University, Nanning, Guangxi 530021, China; ^2^ Guangxi Medical University, Nanning, Guangxi 530021, China

**Keywords:** body mass index, vital capacity, college students, Zhuang nationality, China

## Abstract

**Objective:**

Our study is to evaluate the association between body mass index (BMI) and vital capacity of college students of Zhuang Nationality in China.

**Methods:**

463 college students of Zhuang Nationality from Guangxi Medical University were selected. Basic information, body composition and vital capacity of college students were measured. According to the level of BMI, college students were divided into four groups (BMI<18.5, 18.5≤BMI<23.9, 23.9≤BMI<27.9 and BMI≥27.9). Multivariate logistic regression analysis was performed to assess the association between BMI and vital capacity.

**Results:**

In male college students, there was no significant difference in vital capacity between the four groups (3029.54±869.25, 3347.06±784.54, 3540.00±805.35 and 3966.50±350.2, P=0.0727, respectively). Multivariate regression analysis showed that after adjusting for confounding factors, no significant association was observed between BMI and vital capacity (OR=115.02, 95% CI: -555.58∼785.63; OR=-166.58, 95% CI: -1684.56∼1351.41; OR=-484.01, 95% CI:-3504.53, 2536.51, respectively. BMI<18.5 group served as reference group). In female college students, there was also no significant difference in vital capacity between the four groups (2455.15±574.4, 2555.06±637.03, 2750.33±1224.05 and 2473.00±159.06, P=0.4011, respectively). Multivariate regression analysis showed that after adjusting for confounding factors, no significant association was observed between BMI and vital capacity (OR=-88.88, 95% CI: -333.59∼155.84; OR=20.00, 95% CI: -694.39∼734.39; OR=2.86, 95% CI: -1830.58, 1836.3, respectively. BMI<18.5 group served as reference group).

**Conclusion:**

There was no evidence that BMI is associated with vital capacity in college students of Zhuang Nationality.

## INTRODUCTION

In recent years, overweight and obesity have become global epidemics, and are attributed to changes in living standards not only in developed countries but also in developing countries [1]. Research results have shown that 35% of adults aged ≥20 years were reported to be overweight and 11% were reported to be obese in 2008 year [2–4]. However, obese or overweight people are growing year by year with the rapid economic development and changes in eating habits [5, 6]. In 2012 year, more than 40 million children aged ≥5 years were reported to be overweight or obese all over the world [7, 8].

As is known to all, obesity is a major health issue all over the world. Obesity or/and overweight are a major risk factor for non-communicable disorders as well as cardiovascular disorders [9], such as hypertension [10], diabetes mellitus [11] and dyslipidemia [12], and is closely related to morbidity and mortality. The importance of weight control has been emphasized for the primary and secondary prevention of lifestyle-related disorders on a global scale.

The associations of obesity with reduced pulmonary function and lifestyle-related disorders have received considerable attention. In particular, a close relationship between obesity and restrictive pulmonary dysfunction has been suggested [13–15]. Obesity impacts on many areas of clinical medicine, including pulmonary medicine [16]. Nevertheless, it is debated if obesity is linked to asthma, or whether the obesity, due to its effect of decreasing lung volumes and increasing airway resistance, cause symptoms that simply mimic asthma. Therefore, it is important to understand the relationship between BMI and vital capacity. So far, several previous studies have reported that increased low body weight or BMI is related to reduction of vital capacity, and these results also suggest that the maintenance of adequate body weight may be important for improving the vital capacity [17, 18]. However, evidence is limited with regard to whether BMI is associated with vital capacity among college students of Zhuang Nationality in China.

In our study, we aimed to examine the associations between BMI and vital capacity among college students of Zhuang Nationality in China, and provide theoretical reference for Chinese college students' physical health.

## RESULTS

### Basic characteristics for all college students

Table 1 shows the basic characteristics, including age, body fat, fat, muscle, presumption of bone, body moisture, protein, intracellular fluid, extracellular fluid, body fat percentage, muscle volume ratio, visceral fat area, visceral fat, fat content, waist hip ratio, basal metabolism, energy metabolism, swelling index, trunk muscle the amount of the left upper limb muscles, weight, left lower limb muscle mass, right upper limb muscle, right lower limb muscle mass, trunk fat, trunk fat ratio, left upper limb fat, the left upper limb left lower limb fat, left leg fat, right upper limb fat volume, right upper extremity fat percentage and right lower extremity fat mass, for all college students grouping by sex (121 male college students and 342 female college students).

**Table 1:** Baseline clinical characteristics

Gender	Male	Female
N	121	342
Height (cm)	167.95±5.18	156.87±4.75
Age (year)	19.75±1.44	19.97±1.37
Weight (kg)	58.23±9.38	47.64±5.84
BMI (kg/m^2^)	20.58±2.79	19.34±2.21
Lean body weight (kg)	50.46±5.41	36.39±2.86
Fat content (kg)	7.78±5.06	11.27±3.91
Muscle content (kg)	47.83±5.13	34.37±2.61
Presumptive bone mass (kg)	2.63±0.28	2.02±0.25
Body moisture (kg)	35.04±4.27	25.44±2.27
Protein (kg)	12.84±2.08	8.97±0.87
Intracellular fluid (kg)	21.91±3.14	15.78±1.48
Extracellular fluid (kg)	13.16±1.23	9.69±0.90
Body fat percentage (%)	12.58±5.91	23.12±5.30
Muscle volume ratio (%)	94.99±9.16	95.00±6.22
Visceral fat area (cm^2^)	31.08±26.87	13.21±8.81
Visceral fat content (kg)	0.86±0.97	0.78±0.51
Subcutaneous fat content (kg)	6.88±4.12	10.49±3.44
Waist to hip ratio (%)	0.86±0.04	0.78±0.03
Basal metabolism (kcal/d)	1460.08±164.18	1130.53±89.32
Total energy metabolism (kcal/d)	2168.21±243.82	1678.86±132.64
Edema index (%)	0.36±0.01	0.36±0.01
Trunk muscle mass (kg)	23.99±2.58	17.45±1.68
Left upper limb muscle mass (kg)	2.46±0.33	1.57±0.67
Left lower limb muscle mass (kg)	9.37±1.17	6.91±0.56
Right upper limb muscle mass (kg)	2.54±0.30	1.55±0.18
Right lower limb muscle mass (kg)	9.57±1.17	7.00±0.51
Trunk fat mass (kg)	4.10±3.13	5.00±2.32
Trunk fat rate (%)	12.92±7.37	20.56±6.87
Left upper limb fat mass (kg)	0.33±0.17	0.46±0.27
Left upper extremity fat percentage (%)	10.08±4.56	20.17±5.90
Left lower limb fat mass (kg)	1.57±0.81	2.73±0.63
Left lower extremity fat rate (%)	12.93±4.68	26.78±3.89
Right upper limb fat mass (kg)	0.31±0.17	0.42±0.19
Right upper limb fat rate (%)	9.36±4.39	18.67±5.72
Right lower extremity fat mass (kg)	1.58±0.83	2.77±0.63
Right lower extremity fat rate (%)	12.78±4.67	26.77±3.73

Data are presented as mean±SD.

### Basic characteristics for male college students grouping by BMI

Table 2 shows the basic characteristics, including age, body fat, fat, muscle, presumption of bone, body moisture, protein, intracellular fluid, extracellular fluid, body fat percentage, muscle volume ratio, visceral fat area, visceral fat, fat content, waist hip ratio, basal metabolism, energy metabolism, swelling index, trunk muscle the amount of the left upper limb muscles, weight, left lower limb muscle mass, right upper limb muscle, right lower limb muscle mass, trunk fat, trunk fat ratio, left upper limb fat, the left upper limb left lower limb fat, left leg fat, right upper limb fat volume, right upper extremity fat percentage and right lower extremity fat mass, for male college students grouping by BMI. From Table 2, we can see that the basic characteristics of four groups of male college students has statistical difference except for age (P<0.05).

**Table 2:** Basic characteristics for male college students grouping by BMI

BMI	<18.5	>=18.5, <23.9	>=23.9, <27.9	>=27.9	P-value
N	28	79	10	4	
Age (year)	19.57±1.26	19.82±1.53	19.80±1.40	19.50±1.29	0.862
Lean body weight (kg)	45.77±2.63	50.77±4.26	56.05±5.37	63.15±4.65	<0.001
Fat content (kg)	4.16±1.70	7.19±3.04	16.20±3.16	23.77±4.19	<0.001
Muscle content (kg)	43.37±2.50	48.13±4.05	53.15±5.08	59.88±4.42	<0.001
Presumptive bone mass (kg)	2.40±0.14	2.65±0.22	2.90±0.29	3.27 ± 0.24	<0.001
Body moisture (kg)	31.52±2.53	35.59±3.75	37.99±4.83	41.30±5.49	<0.001
Protein (kg)	11.89±0.99	12.58±1.73	15.19±1.89	18.60±1.63	<0.001
Intracellular fluid (kg)	19.53±2.01	22.38±2.90	23.51±3.64	25.20±4.13	<0.001
Extracellular fluid (kg)	12.02±0.54	13.24±0.90	14.52±1.22	16.12±1.41	<0.001
Body fat percentage (%)	8.26±3.32	12.14±4.25	22.24±2.96	27.35±4.83	<0.001
Muscle volume ratio (%)	86.67±3.79	95.35±6.77	106.70±8.30	116.75±9.73	<0.001
Visceral fat area (cm^2^)	10.00±0.00	28.09±15.29	77.90±10.79	119.85±9.77	<0.001
Visceral fat content (kg)	0.29±0.17	0.69±0.48	2.43±0.62	4.42±0.96	<0.001
Subcutaneous fat content (kg)	3.85±1.54	6.45±2.56	13.75±2.54	19.35±3.28	<0.001
Waist to hip ratio (%)	0.83±0.03	0.86 ± 0.03	0.93±0.01	0.98±0.02	<0.001
Basal metabolism (kcal/d)	1318.57±71.22	1466.28±124.31	1641.40±169.38	1875.00±132.93	<0.001
Total energy metabolism (kcal/d)	1958.04±105.78	2177.42±184.61	2437.60±251.44	2784.25±197.20	<0.001
Edema index (%)	0.36±0.01	0.35±0.01	0.36±0.01	0.36±0.01	0.044
Trunk muscle mass (kg)	21.94±1.50	24.13±2.08	26.21±2.60	29.93±3.27	<0.001
Left upper limb muscle mass (kg)	2.22±0.19	2.50±0.32	2.65±0.35	2.77±0.26	<0.001
Left lower limb muscle mass (kg)	8.43±0.54	9.41±0.97	10.64±1.13	12.08±0.68	<0.001
Right upper limb muscle mass (kg)	2.31±0.21	2.58±0.26	2.73±0.35	2.95±0.26	<0.001
Right lower extremity muscle mass (kg)	8.58±0.51	9.61±0.92	11.01±1.21	12.28±0.70	<0.001
Trunk fat mass (kg)	2.07±1.17	3.66±1.94	9.11±2.01	14.22±2.47	<0.001
Trunk fat rate (%)	8.08±4.38	12.23±5.49	24.68±3.96	31.20±5.55	<0.001
Left upper limb fat mass (kg)	0.20±0.07	0.32±0.11	0.62±0.14	0.75±0.17	<0.001
Left upper extremity fat percentage (%)	6.56±2.36	9.83±3.26	17.82±2.45	20.30±5.35	<0.001
Left lower limb fat mass (kg)	0.92±0.28	1.51±0.48	2.91±0.55	4.00±0.87	<0.001
Left lower extremity fat rate (%)	9.17±2.93	12.79±3.37	20.33±2.13	23.70±4.50	<0.001
Right upper limb fat mass (kg)	0.18±0.07	0.30±0.11	0.60±0.12	0.78±0.21	<0.001
Right upper limb fat rate (%)	5.99±2.23	9.11±3.17	16.88±2.25	19.20±5.02	<0.001
Right lower extremity fat mass (kg)	0.90±0.27	1.52±0.47	3.00±0.56	4.12±0.75	<0.001
Right lower extremity fat rate (%)	8.86±2.81	12.64±3.22	20.30±2.31	24.00±3.96	<0.001

Data are presented as mean±SD.

### Basic characteristics for female college students grouping by BMI

Table 3 shows the basic characteristics, including age, body fat, fat, muscle, presumption of bone, body moisture, protein, intracellular fluid, extracellular fluid, body fat percentage, muscle volume ratio, visceral fat area, visceral fat, fat content, waist hip ratio, basal metabolism, energy metabolism, swelling index, trunk muscle the amount of the left upper limb muscles, weight, left lower limb muscle mass, right upper limb muscle, right lower limb muscle mass, trunk fat, trunk fat ratio, left upper limb fat, the left upper limb left lower limb fat, left leg fat, right upper limb fat volume, right upper extremity fat percentage and right lower extremity fat mass, for male college students grouping by BMI. From Table 3, we can see that the basic characteristics of four groups of female college students has statistical difference except for age (P<0.05).

**Table 3:** Basic characteristics for female college students grouping by BMI

BMI	<18.5	>=18.5, <23.9	>=23.9, <27.9	>=27.9	P-value
N	109	221	9	3	
Age (year)	20.12±1.41	19.91±1.35	19.56±1.42	20.33±0.58	0.442
Lean body weight (kg)	34.71±2.25	37.01±2.66	40.28±3.64	40.17±1.02	<0.001
Fat content (kg)	7.94±1.90	12.24±2.43	21.78±2.31	29.90±3.65	<0.001
Muscle content (kg)	32.83±2.06	34.93±2.43	37.91±3.33	37.80±0.96	<0.001
Presumptive bone mass (kg)	1.88±0.20	2.08±0.24	2.37±0.31	2.33±0.06	<0.001
Body moisture (kg)	23.85±1.55	25.97±1.96	29.21±2.50	32.13±0.81	<0.001
Protein (kg)	9.02±0.70	9.00±0.86	8.72±1.10	5.73±0.12	<0.001
Intracellular fluid (kg)	14.94±1.09	16.07±1.40	17.41±1.69	19.23±0.99	<0.001
Extracellular fluid (kg)	8.94±0.54	9.93±0.66	11.81±0.92	12.93±0.29	<0.001
Body fat percentage (%)	18.42±3.52	24.69±3.56	35.11±2.29	42.60±3.44	<0.001
Muscle volume ratio (%)	91.04±4.77	96.37±5.30	102.41±8.73	116.10±2.04	<0.001
Visceral fat area (cm^2^)	10.09±0.96	12.81±5.93	43.78±10.31	64.17±13.16	<0.001
Visceral fat content (kg)	0.45±0.18	0.85±0.30	2.37±0.54	3.67±0.91	<0.001
Subcutaneous fat content (kg)	7.48±1.74	11.41±2.14	19.42±1.77	26.20±2.74	<0.001
Waist to hip ratio (%)	0.75±0.02	0.79±0.02	0.84±0.02	0.87±0.02	<0.001
Basal metabolism (kcal/.d)	1066.97±62.99	1152.64±77.18	1292.78±101.56	1324.33±24.83	<0.001
Total energy metabolism (kcal/.d)	1584.46 ± 93.52	1711.71±114.62	1919.67±150.89	1966.33±36.95	<0.001
Edema index (%)	0.35±0.01	0.36±0.01	0.37±0.01	0.37±0.01	<0.001
Trunk muscle mass (kg)	16.68±1.39	17.77±1.66	19.06±2.18	17.27±0.38	<0.001
Left upper limb muscle mass (kg)	1.46±0.60	1.61±0.71	1.76±0.17	2.00±0.00	0.127
Left lower limb muscle mass (kg)	6.65±0.48	6.99±0.53	7.63±0.56	8.23±0.51	<0.001
Right upper limb muscle mass (kg)	1.42±0.13	1.59±0.15	1.81±0.23	2.07±0.06	<0.001
Right lower extremity muscle mass (kg)	6.72±0.40	7.09±0.47	7.74±0.50	8.30±0.40	<0.001
Trunk fat mass (kg)	3.10±1.18	5.54±1.53	11.41±1.73	15.10±2.26	<0.001
Trunk fat rate (%)	14.62±4.80	22.52±4.69	36.02±3.19	45.13±4.27	<0.001
Left upper limb fat mass (kg)	0.31±0.18	0.50±0.24	0.94±0.12	1.60±0.17	<0.001
Left upper extremity fat percentage (%)	15.22±4.08	21.78±4.13	33.28±2.35	42.17±3.59	<0.001
Left lower limb fat mass (kg)	2.17±0.32	2.90±0.37	4.29±0.28	5.83±0.55	<0.001
Left lower extremity fat rate (%)	23.34±2.86	27.98±2.68	34.56±1.31	39.97±2.35	<0.001
Right upper limb fat mass (kg)	0.27±0.08	0.46±0.11	0.90±0.10	1.57±0.15	<0.001
Right upper limb fat rate (%)	13.56±3.64	20.35±3.77	31.73±2.59	41.20±3.32	<0.001
Right lower extremity fat mass (kg)	2.21±0.32	2.94±0.37	4.34±0.28	5.90 ± 0.52	<0.001
Right lower extremity fat rate (%)	23.44±2.65	27.93±2.52	34.44±1.20	39.87±2.50	<0.001

Data are presented as mean±SD.

### Multiple regression analysis

**Figure 1:**
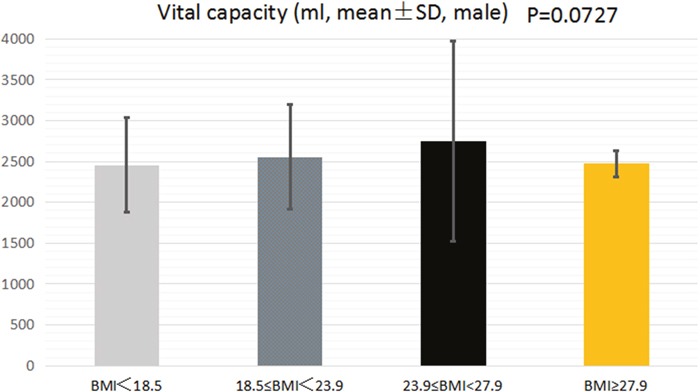
The association between BMI and VC of male college students

Table 4 shows the association between BMI and VC of college students. In male college students, there was no significant difference in vital capacity between the four groups (3029.54±869.25, 3347.06±784.54, 3540.00±805.35 and 3966.50±350.2, P=0.0727, respectively) (Figure 1). Body composition, such as body fat, fat, muscle, presumption of bone, body moisture, protein and intracellular fluid, may have an effect on lung capacity. Therefore, multivariate regression analysis was performed to adjust confounding factors. Multivariate regression analysis showed that after adjusting for confounding factors, no significant association was observed between BMI and vital capacity (OR=115.02, 95% CI: -555.58∼785.63; OR=-166.58, 95% CI: -1684.56∼1351.41; OR=-484.01, 95% CI:-3504.53, 2536.51, respectively. BMI<18.5 group served as reference group).

**Table 4:** Multiple regression analysis

Exposure	Male	Female
Non-adjusted		
BMI		
<18.5	ref	ref
>=18.5, <23.9	317.53 (−26.72, 661.78) 0.0732	99.91 (−46.11, 245.94) 0.1808
>=23.9, <27.9	510.46 (−66.16, 1087.09) 0.0854	295.19 (−137.51, 727.88) 0.1821
>=27.9	936.96 (100.31, 1773.61) 0.0301	17.85 (−712.29, 748.00) 0.9618
Adjust I		
BMI		
<18.5	ref	ref
>=18.5, <23.9	291.47 (−48.9, 631.84) 0.0960	106.19 (−40.09, 252.46) 0.1557
>=23.9, <27.9	486.77 (−82.29, 1055.83) 0.0963	312.42 (−120.88, 745.72) 0.1585
>=27.9	944.37 (119.29, 1769.44) 0.0268	11.31 (−718.41, 741.03) 0.9758
Adjust II		
BMI		
<18.5	ref	ref
>=18.5, <23.9	115.02 (−555.58, 785.63) 0.7376	-88.88 (−333.59, 155.84) 0.4771
>=23.9, <27.9	-166.58 (−1684.56, 1351.41) 0.8302	20.00 (−694.39, 734.39) 0.9563
>=27.9	-484.01 (−3504.53, 2536.51) 0.7543	2.86 (−1830.58, 1836.3) 0.9976

Results are presented as OR (95% CI) P value.

Non-adjusted model adjust for: none.

Adjust I model adjust for: age.

Adjust II model adjust for: age; body fat; fat; muscle; presumption of bone; body moisture; protein; intracellular fluid; extracellular fluid; body fat percentage; muscle volume ratio; visceral fat area; visceral fat; fat content; waist hip ratio; basal metabolism; energy metabolism; swelling index; trunk muscle the amount of the left upper limb muscles; weight; left lower limb muscle mass; right upper limb muscle; right lower limb muscle mass; trunk fat; trunk fat ratio; left upper limb fat; the left upper limb left lower limb fat; fat; left leg fat; right upper limb fat Volume; right upper extremity fat percentage; right lower extremity fat mass.

**Figure 2:**
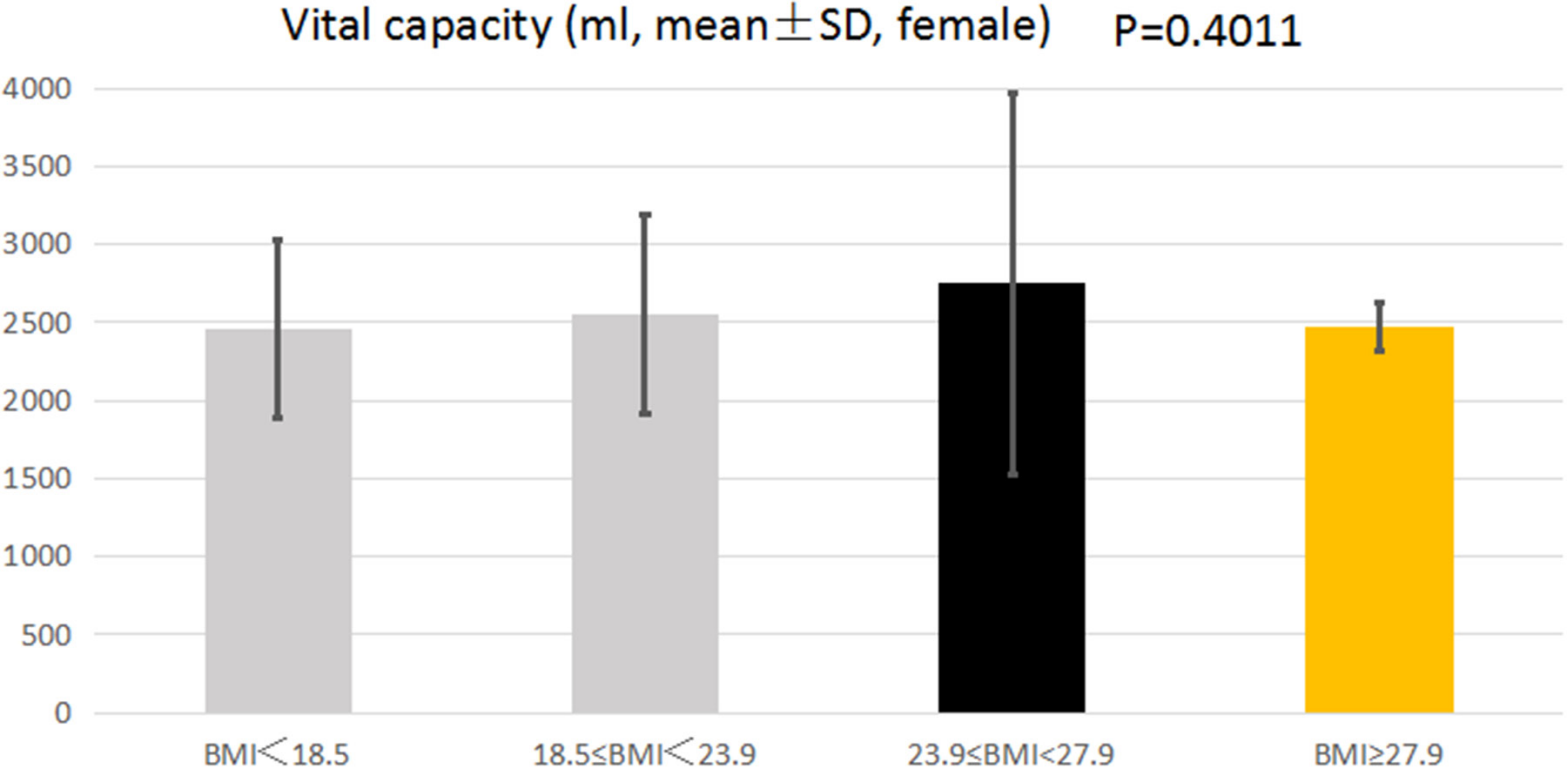
The association between BMI and VC of female college students

In female college students, there was also no significant difference in vital capacity between the four groups (2455.15±574.4, 2555.06±637.03, 2750.33±1224.05 and 2473.00±159.06, P=0.4011, respectively) (Figure 2). Body composition, such as body fat, fat, muscle, presumption of bone, body moisture, protein and intracellular fluid, may have an effect on lung capacity. Therefore, multivariate regression analysis was performed to adjust confounding factors. Multivariate regression analysis showed that after adjusting for confounding factors, no significant association was observed between BMI and vital capacity (OR=-88.88, 95% CI: -333.59∼155.84; OR=20.00, 95% CI: -694.39∼734.39; OR=2.86, 95% CI: -1830.58, 1836.3, respectively. BMI<18.5 group served as reference group).

## DISCUSSION

In our study, we carried out a cross-sectional study to evaluate the association between BMI and VC of college students of Zhuang Nationality in China. 463 college students were recruited in this study, and multivariate logistic regression analysis was performed to adjust confounding factors. Our result found that there was no significant difference in vital capacity between the four groups (underweight, normal BMI, overweight and obese) in male and female college students (all P>0.05). In addition, after multivariate regression analysis was conducted, no significant association was observed between BMI and VC in male and female college students. Therefore, the results from our study suggest that there was no evidence that BMI is associated with VC in college students of Zhuang Nationality in china.

Breathing is an essential function in lung function, which can hinder quality of life and performance in activities of daily living. To maintain respiratory homeostasis, the structures that compose the respiratory system need to work in equilibrium. It means that the lungs should be ventilated and the gases should diffuse through the alveolar-capillary barrier. So far, the efficient method to estimate lung function of college student is by determining VC, which offer information essential for the characterization of the pathophysiological state resulting from abnormalities in the pulmonary-ventilatory processes [19]. Research findings have pointed out that there are many factors that can affect VC, such as weight [20], bone mineral density [21], chronic obstructive pulmonary disease [22], BMI [17], hypertension [23] and diabetes [24]. However, few studies have been done on body mass index and vital capacity, especially in college students. Fukahori S and his colleague [25] conducted a study to evaluate the association between BMI and forced expiratory volume in 1 second/forced vital capacity in a population with a relatively low prevalence of obesity, and 1231 patients were recruited in this research. The result of this study found that BMI was positively correlated with forced expiratory volume in 1 second (FEV (1))/forced vital capacity (FVC) in men and with maximum mid-expiratory flow (MMF) in all subjects. After adjusting for related factors using multiple regression analysis, a significant positive correlation between BMI and FEV (1)/FVC was identified for all subjects. Finally, the authors pointed out that high BMI may be inappropriate as a predictor of obstructive lung dysfunction, particularly in populations with a low prevalence of obesity. In 2014 year, Melo LC and his colleague [26] carried out a systematic review to assess whether obesity is associated with lung function. Nine studies were selected by selecting publications in the science databases MEDLINE and LILACS. The result showed that the obese individuals presented with a reduction in lung volume and capacity as compared to healthy individuals, which means that the presence of a restrictive respiratory pattern associated with obesity. The results of these studies are difference from our results.

At present, there are several potential mechanisms by which BMI might lead to reduce VC, which was broadly divided into mechanical and inflammatory [27]. As BMI continues to rise, the fat content rises gradually, Intra-peritoneal fat deposits and accumulation may impede the descent of the diaphragm during inspiration, which would affect the lung's breathing function [28]. In addition, an increase in abdominal fat volume can reduce the expiratory reserve volume, by displacing the diaphragm upward and reducing functional volume in the thoracic cavity [29]. Furthermore, the deposition of fat on the chest wall have negative effects on the expansion and excursion of the rib cage, through a direct loading effect or by altering intercostal muscle function [30]. Chlif M [31] carried out a study recently and pointed out that during exercise, obese volunteers show a decrease in inspiratory muscle activity as a result of reduced inspiratory strength and increased ventilatory drive. Some researchers also pointed out that increased BMI was associated with markers of systemic and vascular inflammation, such as C-reactive protein and leptin [32–34]. The inflammatory factors, such as TNF-α, IL-1β, IL-6 and TG F-β, may exert local effects in lung tissue, and lead to subtle reductions in airway diameter.

In our study, the relationship between BMI and VC in college students of Zhuang Nationality is not obvious. With the rise of BMI, VC does not show a downward trend. After adjusting confounding factors by using multivariate regression analysis, the results did not change significantly. Besides, in our analysis stratified by gender, the results also showed no significant change. Our results are inconsistent with previous studies mentioned above. There may be several reasons for this phenomenon. First, the subjects of this study are Chinese, and the difference in population may lead to this phenomenon; second, college students are a healthy group with few diseases. Thus eliminating the influence of diseases (such as hypertension, coronary heart disease, diabetes mellitus), and then further study the independent relationship between BMI and VC. The Zhuang nationality is a special race in Guangxi, China, and few studies have reported this population. Perhaps racial differences can also lead to inconsistent results. Generally, our result is the first study on the association between BMI and VC in college students of Zhuang Nationality. On the other hand, our research also suggests that the mechanisms put forward by former scholars may not apply to college students, and new research mechanisms need to be explored for college students.

### Limitations

A number of limitations should be considered when interpreting findings from this study. First, our analysis was a cross-sectional study, which makes the inference of causality difficult. It remains to be seen whether reducing BMI will improve VC in this cohort. Although confounding factors, such as age, body fat, fat and muscle, were controlled by using regression analysis, we cannot completely remove residual confounding due to measurement error. Second, our research subjects are Chinese college students, especially Zhuang college students. Therefore, the findings of our study do not necessarily apply to other populations, such as African college students, European college students and American college students due to environmental factors and dietary factors, etc.

In summary, we found that there was no evidence that BMI is associated with VC in college students of Zhuang Nationality. However, due to small sample size and the presence of potential confounding bias, further studies are required to confirm the conclusion, and to elucidate the mechanisms by which BMI influences VC, particularly in college students coming from different regions and country.

## MATERIALS AND METHODS

**Figure 3:**
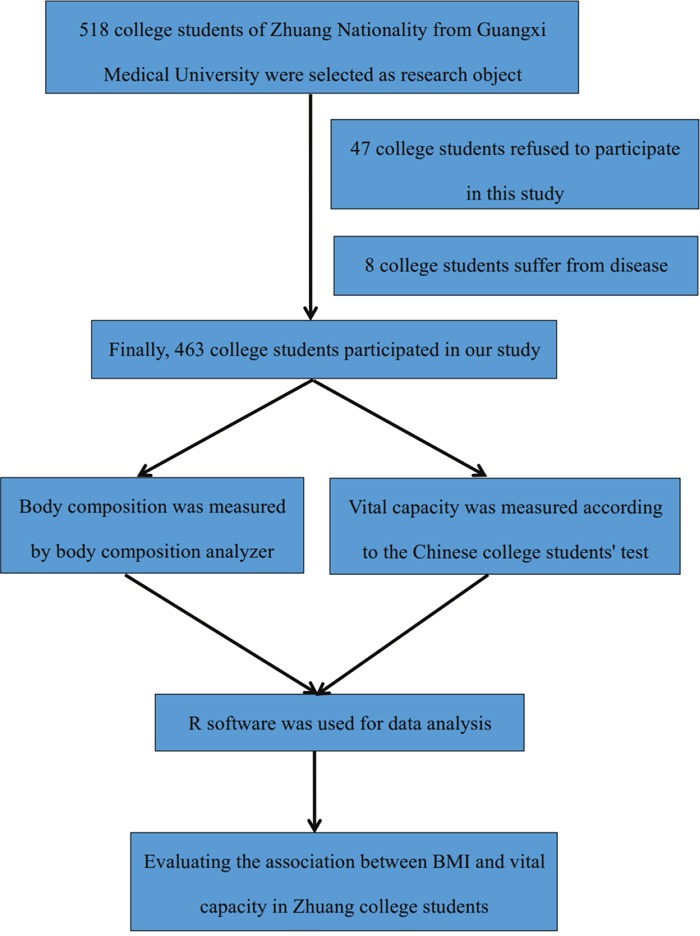
Study population flow chart

518 college students of Zhuang Nationality from Guangxi Medical University were selected as research object. Inclusion criteria: 1) Age≥18 years; 2) No suffer from disease in the past 1 year, such as tuberculosis, osteoporosis, liver and kidney disease and so on; 3) All college students are aware of this study and written informed consent was obtained from all college students. Exclusion criteria: 1) Non Zhuang college students; 2) Refused to participate in this study; 3) Suffer from a disease in the past 1 year. According to the inclusion criteria and exclusion criteria, 55 college students were excluded from our study (47 college students refused to participate in this study and 8 college students suffer from disease). Flow chart is shown in Figure 3.

### Study protocol

Our research has obtained the approval of the Ethics Committee of Guangxi Medical University, and all patients provided written informed consent. All patients were fully informed about the study protocol and signed informed consent before this study.

### BMI

Height and body weight, were measured using standardized methods to nearest 0.1 cm or 0.1 kg in subjects wearing light clothing, and BMI was calculated by dividing body weight (kg) by height squared (m^2^). All college students were divided into five different BMI categories: underweight, <18.5kg/m^2^; normal BMI, 18.5-23.9kg/m^2^; overweight, 23.9-27.9kg/m^2^; obese, BMI greater than or equal to 27.9 kg/m^2^ (including male college students and female college students).

### Body composition

Body composition, including body fat, fat, muscle, presumption of bone, body moisture, protein, intracellular fluid, extracellular fluid, body fat percentage, muscle volume ratio, visceral fat area, visceral fat, fat content, waist hip ratio, basal metabolism, energy metabolism, swelling index, trunk muscle the amount of the left upper limb muscles, weight; left lower limb muscle mass, right upper limb muscle, right lower limb muscle mass, trunk fat, trunk fat ratio, left upper limb fat, the left upper limb left lower limb fat, fat; left leg fat, right upper limb fat Volume, right upper extremity fat percentage and right lower extremity fat mass, were measured by tetrapolar bioelectrical impedance analysis (InBody 3.0, Biospace, Seoul, Korea). All college students wear light clothes when they are measuring, without shoes.

### Vital capacity

The vital capacity (VC) measures the maximum amount of air that can be inhaled or exhaled during a respiratory cycle. It is the sum of the expiratory reserve volume, tidal volume, and inspiratory reserve volume. The inspiratory capacity (IC) is the amount of air that can be inhaled after the end of a normal expiration. It is, therefore, the sum of the tidal volume and inspiratory reserve volume. The total lung capacity (TLC) is a measurement of the total amount of air that the lung can hold. It is the sum of the residual volume, expiratory reserve volume, tidal volume, and inspiratory reserve volume. The unit of VC is milliliter (ml).

### Statistical analyses

All descriptive data are presented as means and standard deviations or as numbers and percentages. The baseline characteristics for descriptive data were compared using Student's t test, and categorical variables were analyzed using the *X*^2^ test. We calculated odds ratio (OR) and 95% confidence intervals (CI) for the associations between body mass index (BMI) and vital capacity.

Non-adjusted models was conduct to adjust for none based on multiple regression analysis; Adjust I model was conduct to adjust for age based on multiple regression analysis; Adjust II model was conduct to adjust for age; body fat; fat; muscle; presumption of bone; body moisture; protein; intracellular fluid; extracellular fluid; body fat percentage; muscle volume ratio; visceral fat area; visceral fat; fat content; waist hip ratio; basal metabolism; energy metabolism; swelling index; trunk muscle the amount of the left upper limb muscles; weight; left lower limb muscle mass; right upper limb muscle; right lower limb muscle mass; trunk fat; trunk fat ratio; left upper limb fat; the left upper limb left lower limb fat; fat; left leg fat; right upper limb fat Volume; right upper extremity fat percentage; right lower extremity fat mass based on multiple regression analysis.

All of the analyses were performed with the statistical software packages R (http://www.R-project.org, The R Foundation). A two-sided significance level of 0.05 was used to evaluate statistical significance.
